# Reply to Bhowmik et al.: Democratic climate action and studying extreme climate risks are not in tension

**DOI:** 10.1073/pnas.2216034119

**Published:** 2022-11-02

**Authors:** Luke Kemp, Chi Xu, Joanna Depledge, Kristie L. Ebi, Goodwin Gibbins, Timothy A. Kohler, Johan Rockström, Marten Scheffer, Hans Joachim Schellnhuber, Will Steffen, Timothy M. Lenton

**Affiliations:** ^a^Centre for the Study of Existential Risk, University of Cambridge, Cambridge CB2 1SB, United Kingdom;; ^b^Darwin College, University of Cambridge, Cambridge CB3 9EU, United Kingdom;; ^c^School of Life Sciences, Nanjing University, Nanjing 210023, China;; ^d^Cambridge Centre for Environment, Energy and Natural Resource Governance, University of Cambridge, Cambridge CB2 3QZ, United Kingdom;; ^e^Center for Health and the Global Environment, University of Washington, Seattle, WA 98195;; ^f^Future of Humanity Institute, University of Oxford, Oxford OX2 0DJ, United Kingdom;; ^g^Department of Anthropology, Washington State University, Pullman, WA 99164-4910;; ^h^Santa Fe Institute, Santa Fe, NM 87501;; ^i^Cluster of Excellence ROOTS, Christian-Albrechts-Universität, 24118 Kiel, Germany;; ^j^Potsdam Institute for Climate Impact Research, 14473 Potsdam, Germany;; ^k^Department of Environmental Sciences, University of Wageningen, 6708PB Wageningen, The Netherlands;; ^l^Earth System Science Department, Tsinghua University, 100190 Beijing, China;; ^m^Fenner School of Environment and Society, The Australian National University, Canberra, ACT 2601, Australia;; ^n^Global Systems Institute, University of Exeter, Exeter EX4 4QE, United Kingdom

We thank Bhowmik et al. for their letter, “From Climate Endgame to Climate Long Game” (1), in response to our manuscript “Climate Endgame: Exploring catastrophic climate change scenarios” (2). We agree that extreme climate risks are currently understudied, and that it is vital and possible to understand them.

We strongly support their call for democratic, inclusive governance and participatory action research. Indeed, this is why we suggested, in “Climate Endgame,” that catastrophic climate risk assessments should be “fed into open deliberative democratic methods that provide a fair, inclusive, and effective approach to decision-making” (2). Doing so enhances fairness, improves collective judgement (3, 4), and provides democratic safeguards (5).

The proposed “Climate Long Game” of Bhowmik et al. (1) is a useful complement to the Climate Endgame research agenda and can be used in tandem. They are neither alternatives nor in tension.

We diverge from Bhowmik et al. (1) in their contention that studying catastrophic climate risks could portray the threats as inevitable and lead to paralysis. This is a common and mistaken argument that hinges on a fundamental misunderstanding of risk. Risk is probabilistic. Humans have agency, through policies and interventions promoting mitigation, adaptation, and resilience, to alter both the probability and potential consequences of exposure to extreme climate-related hazards. Their proposed agenda and ours share an interest in promoting such agency and channeling it productively.

There is no strong evidence that discussing extreme risks will cause fatalism. As we note in “Climate Endgame” (2), metaanalyses of hopeful vs. fearful messaging have mixed results (6, 7). Both positive and negative emotions can be used in climate communications to change intentions and actions, depending on the specific audience being targeted (8). One review found that negative affect (concern about climate change) toward climate change was the biggest predictor of willingness to engage in individual climate mitigation action (9). Indeed, even one of the articles referenced by Bhowmik et al. (1) notes that “worry,” not “hope,” is the single greatest predictor of higher support of climate action (10). We should also think beyond the false dichotomy of “hope” vs. “fear” in messaging. For instance, there are robust findings in social psychology on the need for selecting the most effective messengers and frames based on the audience and local context (11).

Scholars have a duty to conduct comprehensive assessments of the broad range of potential risks to human and natural systems associated with our changing climate, and to communicate these honestly and clearly.

In other domains such as finance and medicine, society expects a full diagnostic to address risk. What we are proposing is a full planetary diagnostic. Together with Bhowmik et al. (1), we advocate that this diagnostic and our responses should be inclusive, deliberative, and democratic.

Our call for the Intergovernmental Panel on Climate Change to prepare a special report on this topic could be a powerful avenue for triggering wide-ranging research, including through participatory approaches. We wish Bhowmik et al. (1) every success in pursuing their research agenda and look forward to finding opportunities for synergies between our efforts.
